# Melatonin as a Potential Neuroprotectant: Mechanisms in Subarachnoid Hemorrhage-Induced Early Brain Injury

**DOI:** 10.3389/fnagi.2022.899678

**Published:** 2022-04-29

**Authors:** Chengyan Xu, Zixia He, Jiabin Li

**Affiliations:** ^1^Department of Neurosurgery, The Children’s Hospital Zhejiang University School of Medicine, National Clinical Research Center for Child Health, Hangzhou, China; ^2^Department of Outpatient, The Children’s Hospital Zhejiang University School of Medicine, National Clinical Research Center for Child Health, Hangzhou, China; ^3^Department of Pharmacy, The Children’s Hospital Zhejiang University School of Medicine, National Clinical Research Center for Child Health, Hangzhou, China

**Keywords:** subarachnoid hemorrhage, early brain injury, melatonin, mechanism, apoptosis, inflammation, vasospasm, oxidative stress

## Abstract

Subarachnoid hemorrhage (SAH) is a common cerebrovascular disease with high mortality and disability rates. Despite progressive advances in drugs and surgical techniques, neurological dysfunction in surviving SAH patients have not improved significantly. Traditionally, vasospasm has been considered the main cause of death and disability following SAH, but anti-vasospasm therapy has not benefited clinical prognosis. Many studies have proposed that early brain injury (EBI) may be the primary factor influencing the prognosis of SAH. Melatonin is an indole hormone and is the main hormone secreted by the pineal gland, with low daytime secretion levels and high nighttime secretion levels. Melatonin produces a wide range of biological effects through the neuroimmune endocrine network, and participates in various physiological activities in the central nervous system, reproductive system, immune system, and digestive system. Numerous studies have reported that melatonin has extensive physiological and pharmacological effects such as anti-oxidative stress, anti-inflammation, maintaining circadian rhythm, and regulating cellular and humoral immunity. In recent years, more and more studies have been conducted to explore the molecular mechanism underlying melatonin-induced neuroprotection. The studies suggest beneficial effects in the recovery of intracerebral hemorrhage, cerebral ischemia-reperfusion injury, spinal cord injury, Alzheimer’s disease, Parkinson’s disease and meningitis through anti-inflammatory, antioxidant and anti-apoptotic mechanisms. This review summarizes the recent studies on the application and mechanism of melatonin in SAH.

## Introduction

Subarachnoid hemorrhage (SAH) is one of the common cerebrovascular diseases, and its incidence varies in different countries and regions; the overall incidence is about 6/100,000 people per year (Etminan et al., 2019). The incidence of SAH gradually increases with age. Due to the advances in medical and surgical techniques, the mortality rate of SAH has decreased over the past few decades but still remains prevalent (Macdonald and Schweizer, 2017). In addition, 33% of SAH survivors experienced a high disability rate and required long-term care (Al-Khindi et al., 2010). Vasospasm has traditionally been considered a major cause of death and disability post-SAH as it can induce delayed cerebral ischemia. Therefore, most of the studies in the past decades have focused on reducing vasospasm with the aim of improving outcomes of SAH patients (Kassell et al., 1985; Macdonald and Weir, 1991; Cook, 1995; Crowley et al., 2008; Macdonald, 2016; Etminan and Macdonald, 2021). However, delayed cerebral ischemia still occurs in a considerable proportion of SAH patients, even if the cerebral vasospasm is reversed in the early stage. In addition, not all cerebral infarction after SAH is caused by cerebral vasospasm in clinical practice. Cerebral infarction may occur immediately after the occurrence of SAH in some patients, without any cerebral angiography findings (Naidech et al., 2006). Several recent large clinical trials have suggested that treating vasospasm does not significantly improve patient outcomes (Macdonald et al., 2008, 2011; Shen et al., 2013). It is suggested that there may be other mechanisms of injury affecting the prognosis of SAH patients. Recently, the concept of early brain injury (EBI) was proposed, which refers to brain injury occurring within 72 h after the occurrence of SAH (Cahill and Zhang, 2009). EBI is an event with complex pathophysiological changes, including increased intracranial pressure, decreased cerebral blood flow, and direct hematoma toxicity to the brain tissue. These subsequently lead to the destruction of the blood-brain barrier (BBB), oxidative stress injury, cellular death, inflammatory response, microcirculation dysfunction and mitochondrial disorder, causing neurologic injury and poor outcome after SAH (Cahill et al., 2006; Ostrowski et al., 2006; Sehba et al., 2012; Fujii et al., 2013; Ji and Chen, 2016). Therefore, exploring efficient therapeutic methods targeting EBI is essential in treating SAH.

Melatonin is a type of neuroendocrine hormone produced by the pineal gland, with low daytime levels and high nighttime levels (Tordjman et al., 2017; Cardinali, 2021). It participates in regulating the immune, reproductive, endocrine and central nervous systems, and has attracted widespread attention due to its strong anti-inflammatory and antioxidant properties (Majidinia et al., 2018; He et al., 2021; Kvetnoy et al., 2022). Recent studies have shown that melatonin exerts a neuroprotective role in many neurological diseases such as stroke (Lee et al., 2007; Tai et al., 2010), trauma injury (Samantaray et al., 2009; Osier et al., 2018), neurodegenerative diseases (Alamdari et al., 2021; Roy et al., 2022), and spinal cord injury (Hong et al., 2010; Zhang et al., 2018). The mechanisms involved include anti-inflammation, anti-apoptosis, anti-oxidative stress, and BBB protection. In addition, numerous studies have been carried out on the role and mechanism of melatonin in SAH, and the results established that melatonin can improve brain injury after SAH through a variety of mechanisms, thus alleviating neurological impairment and improving prognosis (Martinez-Cruz et al., 2002). This study summarizes the current literature on melatonin treatment in EBI after SAH (Table 1). It explores the relevant mechanism of melatonin-induced neuroprotection, providing a theoretical basis for the experimental study and clinical application of melatonin treatment for SAH.

**TABLE 1:** Neuroprotection of melatonin treatment in SAH.

Therapeutic paradigm	Main findings	References
5 mg or 10 mg/kg, injected into the cisterna magna at 1 h before SAH.	Melatonin prevents focal cerebellum injury by induction of HO-1. The antioxidant capability of melatonin is higher than vitamin E.	Martinez-Cruz et al., 2002
5 mg/kg, intraperitoneally injection every 12 h for 48 h, start at 2 h after SAH.	Melatonin prevents SAH-induced vasospasm and apoptosis of endothelial cells of vessels.	Aydin et al., 2005
15 mg or 150 mg/kg, intraperitoneally injection at 2 h after SAH.	High doses of melatonin (150 mg/kg) reduce brain edema and mortality after SAH.	Ayer et al., 2008b
15 mg or 150 mg/kg, intraperitoneally injection at 2 h after SAH.	High doses of melatonin (150 mg/kg) reduce brain edema and mortality after SAH, which is unrelated to oxidative stress inhibition.	Ayer et al., 2008a
10 mg/kg, intraperitoneally injection immediately after SAH, then daily for 2 days.	Melatonin alleviates oxidative stress, restores BBB permeability and reduces brain edema after SAH.	Ersahin et al., 2009
20 mg/kg, intraperitoneally injection at 6 h after SAH, twice daily for 5 days.	Melatonin alleviates cerebral vasospasm by elevating NO levels in serum and downregulating the levels of arginase and oxidative stress in the brain.	Aladag et al., 2009
5 mg/kg, intraperitoneally injection every 12 h for 120 h, start immediately after SAH.	Melatonin attenuates inflammatory response and oxidative stress in the spasmodic artery and alleviates cerebral vasospasm post-SAH.	Fang et al., 2009
150 mg/kg, intraperitoneally injection at 2 h and 24 h after SAH	Melatonin attenuates EBI *via* activating the Nrf2-ARE pathway and regulating oxidative stress by inducing antioxidant and detoxifying enzymes.	Wang et al., 2012
150 mg/kg, intraperitoneally injection at 2 and 24 h after SAH	Melatonin exerts neuroprotection through anti-oxidative and anti-inflammatory signaling pathways following SAH.	Wang et al., 2013
150 mg/kg, intraperitoneally injection at 2 h after SAH.	Melatonin improves the neurological outcome by reducing neuronal apoptosis and enhancing autophagy *via* a mitochondrial pathway.	Chen et al., 2014b
150 mg/kg, intraperitoneally injection at 2 h after SAH.	Melatonin inhibits the degradation of tight junction proteins, attenuates cerebral edema, improves BBB dysfunctions by inhibiting the inflammatory response.	Chen et al., 2014a
150 mg/kg, intraperitoneally injection at 2 h after SAH.	Melatonin attenuates neurogenic pulmonary edema by preventing alveolar-capillary barrier dysfunctions *via* repressing the inflammatory response and reducing apoptosis after SAH.	Chen et al., 2015
150 mg/kg, intraperitoneally injection at 2 h after SAH.	Melatonin attenuates the EBI post-SAH by inhibiting NLRP3 inflammasome-associated apoptosis.	Dong et al., 2016
150 mg/kg, intraperitoneally injection at 2 and 12 h after SAH.	Melatonin attenuates EBI following SAH *via* the MR/Sirt1/NF-κB signaling pathway.	Zhao et al., 2017
150 mg/kg, intraperitoneally injection at 2 h after SAH.	Melatonin exerts a neuroprotective effect after SAHA by inhibiting mitophagy-associated NLRP3 inflammasome.	Cao et al., 2017
150 mg/kg, intraperitoneally injection at 2 and 12 h after SAH.	Melatonin attenuates EBI after SAH by regulating the H19-miR-675-P53 and H19-let-7a-NGF signaling pathways.	Yang et al., 2018b
15 mg or 150 mg/kg, intraperitoneally injection at 2 h after SAH.	Melatonin attenuates SAH-induced EBI by diminishing neuronal apoptosis and autophagy, partially involving the ROS-MST1 pathway.	Shi et al., 2018
150 mg/kg, intraperitoneally injection at 2 and 12 h after SAH.	Melatonin attenuates EBI after SAH by regulating the protein expression of SIRT3.	Yang et al., 2018a
150 mg/kg, intraperitoneally injection at 2 and 12 h after SAH.	Melatonin provides protection against EBI post-SAH by inducing mitophagy and increasing the expression of NRF2.	Sun et al., 2018
Intraperitoneally injection at 2 h after SAH.	Melatonin treatment attenuates EBI following SAH *via* the JAK1/STAT3 signaling pathway.	Li et al., 2019
150 mg/kg, intraperitoneally injection at 2 and 12 h after SAH.	Melatonin ameliorates cerebral vasospasm by regulating the H19/miR-138/eNOS and H19/miR-675/HIF1α signaling pathways.	Hou et al., 2020
50 mg/kg, 150 mg/kg, or 300 mg/kg, intraperitoneally injection at 15 min after SAH.	Melatonin exerts a white matter-protective effect in SAH pathophysiology, possibly by attenuating apoptosis in oligodendrocytes.	Liu et al., 2020
150 mg/kg, intraperitoneally injection at 12 h after SAH.	Melatonin ameliorates delayed brain injury following SAH *via* H19/miR-675/HIF1A/TLR4 signaling pathway.	Xu et al., 2022

## The Effect and Mechanism of Melatonin in Subarachnoid Hemorrhage

### Melatonin and Vasospasm

Cerebral vasospasm is a common complication of SAH, which usually appears around 3 days after SAH, and reaches its peak within 10 days after SAH (Etminan et al., 2011; Gaspard, 2020). Delayed cerebral ischemia caused by cerebral vasospasm leads to cerebral infarction, cerebral hernia and other malignant complications (Kumar et al., 2016; Ikram et al., 2021). In the acute stage following SAH, the nitric oxide (NO)/NO synthase (NOS) system and vasoconstrictor factors may be involved in the occurrence of cerebral vasospasm. The NO/NOS system plays an important role regulating hemodynamics. NO regulates cerebral blood flow and blood pressure by dilating blood vessels, inhibiting platelet aggregation, and diminishing leukocyte adhesion to the intima (Toda et al., 2009; Attia et al., 2015; Vanhoutte, 2018). After SAH onset, decreased NO levels are observed, leading to CBF reduction, cerebral vasoconstriction, and platelet aggregation. In addition, as a form of free radical, NO enters the intima cells, causing damage to mitochondria and blood vessels (Sehba and Bederson, 2011; Crobeddu et al., 2016; Ehlert et al., 2016; Guo et al., 2016). Endothelin-1 (ET-1) is a potential vasoconstrictor mainly released by astrocytes and leukocytes during the early inflammatory response stage after SAH (Cosentino and Katusić, 1994; La and Reid, 1995; Vergouwen et al., 2012). Previous studies found that ET-1 levels in serum and plasma increased within a few minutes after SAH, and the expression of its receptor increased within 48 h (Lin et al., 2004, 2006). The declined levels of NO increases the expression of ET-1, causing continuous vasocontraction and degenerative morphological changes of the vessels. Notably, previous studies mainly focus on the spasms of large blood vessels, while ignoring the microvascular changes. The cerebral microvasculature has been recently identified as an important intervention target after SAH. Changes in the anatomical structure of cerebral microvessels, sufficient to cause functional deficits, are found in the early post-SAH period. After SAH occurrence, constriction of microvessels contributes to intima remodeling, basal lamina degradation, increased vascular permeability, and eventually leads to microcirculation disorders and brain injury (Nagai et al., 1976; Sehba and Friedrich, 2011).

To explore whether melatonin treatment reverses vasospasm in a SAH model, light microscopic measurements of the basilar arteries were performed to illustrate the arterial wall changes (Fang et al., 2009). Melatonin injection simultaneously with SAH or 2 h after SAH both were found to attenuate the constriction of vessels (Aydin et al., 2005). Additionally, melatonin-induced improvement of cerebral vasospasm is associated with increased serum NO levels, decreased arginase levels, and oxidative stress in the brain (Aladag et al., 2009). Furthermore, the potential underlying mechanism of melatonin treatment after SAH was studied. Hou et al. (2020) reported that melatonin ameliorates post-SAH vasospasm by regulating the expression of endothelial nitric oxide synthase (eNOS) and hypoxia-inducible factor-1 (HIF-1α) *via* the H19/miR-138/eNOS/NO and H19/miR-675/HIF-1α signaling pathways. However, the crosstalk of the pathway network is complex, and the exact mechanism behind the anti-vasospasm effect of melatonin needs further research. Recently, a clinical trial provided evidence for the delayed elevation of circulatory daytime melatonin after SAH and described the role of aneurysm location, resulting in high levels during the critical phase (Neumaier et al., 2021). However, the relationship between endogenous melatonin level changes and vasospasm were not discussed. Further clinical trials focusing on the role of endogenous changes and administration of melatonin in vasospasm after SAH may provide more clinical evidence for the clinical application of melatonin in SAH.

### Melatonin and Inflammation

Previous studies have found that in the early SAH stage, erythrocyte degradation products accumulated in the subarachnoid space, activating an inflammatory response and participating in the acceleration of brain injury (Schallner et al., 2015; Zhang et al., 2016). Both experimental studies and clinical trials have demonstrated that the aseptic inflammatory response after SAH can aggravate tissue damage and is an independent predictor of mortality in SAH patients (Pradilla et al., 2010; Muroi et al., 2011; Luo et al., 2021; Devlin et al., 2022). Microglia are innate immune cells of the central nervous system and can be activated rapidly under inflammatory conditions, trauma, or other stimulating factors. Previous studies have confirmed that microglia can be activated within minutes of the occurrence of SAH and contribute to the process of inflammation (Coulibaly and Provencio, 2020; Heinz et al., 2021; Chen et al., 2022). When microglia are activated in a normal physiological state, they can remove harmful substances by phagocytosis. However, when microglia are overactivated, they exacerbate brain injury by releasing pro-inflammatory factors and oxidative metabolites, promoting the activation of neutrophils and macrophages, thus resulting in the destruction of BBB, inflammatory response and neuronal damage (Lucke-Wold et al., 2016; van Dijk et al., 2016; Schneider et al., 2018). Similar to microglia, astrocytes can also synthesize and secrete inflammatory factors (such as cytokines and chemokines) and participate in the inflammatory response of SAH (Gris et al., 2019; Zhang et al., 2021). In addition to inflammatory cells, inflammatory-related proteins such as C-reactive protein, intercellular adhesion molecule (ICAM)-1, high mobility group box 1 (HMGB1), and galectin-3 also play key roles in the inflammatory reaction following SAH (Lin et al., 2007; Sun et al., 2014; Nishikawa and Suzuki, 2018; Mota Telles et al., 2021). Damage-associated molecular patterns (DAMPs) are released by neurons, astrocytes, microglia and endothelial cells in the early stage of SAH, activating local and peripheral immune cells and releasing cytokines to promote an inflammatory response. This process leads to early brain injury (Chaudhry et al., 2018; Lu et al., 2018; Ahmed et al., 2021; Balança et al., 2021). Furthermore, pro-inflammatory cytokines, such as interleukin-1 β (IL-1β), interleukin-6 (IL-6) and tumor necrosis factor -α (TNF-α), can trigger an inflammatory cascade that ultimately leads to the destruction of the BBB, and cause secondary injury post-SAH (Duris et al., 2018; Savarraj et al., 2018; Okada and Suzuki, 2020). Therefore, therapies inhibiting the inflammatory response may alleviate the EBI after SAH, thereby improving the prognosis of patients.

Melatonin has shown anti-inflammatory properties in SAH. It can reduce neuroinflammation and improve axonal hypomyelination by modulating M1/M2 microglia polarization *via* the JAK2-STAT3-telomerase pathway (Zhou et al., 2021). Moreover, melatonin reduces the release of pro-inflammatory mediators (IL-1β, IL-6, and TNF-α, etc.), alleviates the inflammatory response, thus improving secondary brain damage and neurobehavioral dysfunction after SAH (Fang et al., 2009). In addition, inhibition of inflammation by melatonin effectively protects the integrity of the BBB structure and function, and reduces the degree of brain edema (Chen et al., 2014a,^2015^). Wang et al. (2013) found that melatonin markedly decreased the expressions of TLR4 pathway-related agents, such as HMGB1, toll-like receptor 4 (TLR4), myeloid differentiation factor 88 (MyD88), indicating the involvement of the TLR4 pathway in melatonin-induced neuroprotection. Among the inflammatory responses after SAH, NLRP3 inflammasome activation has recently been investigated. NLRP3 inflammasome activation promotes the maturation and secretion of pro-inflammatory cytokines and participates in cell death processes such as pyroptosis. Various studies have revealed that reduction of NLRP3 inflammasome activation exerts strong neuroprotective effects in the acute phase following SAH, which was associated with the downregulation of pro-inflammatory cytokines. Cao et al. (2017) proposed that melatonin is neuroprotective against EBI post-SAH *via* inhibiting mitophagy-associated NLRP3 inflammasome. In addition, Dong et al. (2016) demonstrated that melatonin treatment attenuates brain injury by inhibiting NLRP3 inflammasome-associated apoptosis following SAH. Notably, NLRP3 signal activation is performed by microglia, and reduced NLRP3 is related to decreased white matter injury after melatonin treatment in SAH (Liu et al., 2020).

### Melatonin and Apoptosis

Apoptosis is one of the main pathophysiological processes of EBI, and its degree is closely related to the neurological function recovery after SAH (He et al., 2012; Hong et al., 2014). Previous experiments have shown that apoptosis of neurons begins within 10 min after the occurrence of SAH (Friedrich et al., 2012). The increased intracranial pressure, cerebral edema and oxidative stress induced by SAH can all lead to extensive cell apoptosis, including neurons, glial cells and vascular endothelial cells (Ostrowski et al., 2006; Hasegawa et al., 2011; Shao et al., 2020). Apoptosis after SAH involves many pathways, including the death receptor pathway, mitochondrial pathway, and dependent or independent caspase pathway (Cahill et al., 2006). The mitochondrial pathway is mediated by the Bcl-2 family and manifests as increased permeability of the mitochondrial membrane. Cytochrome c transfers from mitochondria to the cytoplasmic septum and participates in apoptosome assembly with apoptosis protease-activator factor 1 (Apaf-1), thus leading to the activation of caspase-9. Subsequently, caspase-3 is further activated to induce apoptosis (Ceccatelli et al., 2004; Bornstein et al., 2020). The caspase-independent pathway is mainly mediated by the mitochondrial release of apoptosis-inducing factor (AIF). AIF exists in the mitochondrial membrane gap in its normal state, and can be transferred to the nucleus after SAH to induce DNA destruction and cell apoptosis without caspase activation (Lorenzo et al., 1999; Lorenzo and Susin, 2007; Norberg et al., 2010). Death receptors participate in external apoptosis pathways. The expression of Fas and TNF ligands of death receptors are upregulated after SAH, binding to death receptors and activating the caspase cascade (Gorojod et al., 2017; Cha et al., 2019). These pathways interact with each other and participate in the occurrence and regulation of apoptosis after SAH. By intervening on the above pathways, apoptosis may be effectively alleviated, thus improving the neurological injury and promoting the recovery of neurologic function after SAH.

The neuroprotective role of melatonin by diminishing cellular apoptosis in EBI after SAH was investigated. A study published in 2005 first reported the anti-apoptosis effect of melatonin in a SAH rabbit model by reducing the apoptosis of endothelial cells (Aydin et al., 2005). Subsequently, many studies confirmed the role of melatonin in alleviating neuronal apoptosis after SAH, contributing to the amelioration of spatial learning and memory deficits and improvement of therapeutic outcomes (Wang et al., 2012, 2013; Dong et al., 2016; Sun et al., 2018). Moreover, melatonin can reduce oligodendrocyte apoptosis associated with the attenuation of white matter injury. The mechanism of the above anti-apoptosis effects is related to enhancing autophagy, which improves cell apoptosis *via* a mitochondrial pathway (Chen et al., 2014b). In addition, melatonin reduces cellular apoptosis partially *via* the regulation of the melatonin receptor/Sirt1/NF-κB signaling pathway (Zhao et al., 2017), ROS/mammalian sterile 20-like kinase 1 (MST1) pathway (Shi et al., 2018), ROS/SIRT3 pathway (Yang et al., 2018a), and JAK/STAT pathway (Li et al., 2019). Recently, many studies have shown that melatonin abolished apoptosis by regulating microRNAs. Xu et al. (2022) established that melatonin affects HIF-1α and ameliorates delayed brain injury following SAH *via* the H19/miR-675/HIF1A/TLR4 pathway. In contrast, Yang et al. (2018b) demonstrated that long non-coding RNA and microRNA-675/let-7a mediate the protective effect of melatonin against EBI after SAH *via* targeting TP53 and neural growth factor. Notably, melatonin not only reduces cell apoptosis in the brain but also prevents alveolar-capillary barrier dysfunctions *via* repressing cell apoptosis in the lung, thus attenuating neurogenic pulmonary edema after SAH (Chen et al., 2015).

### Melatonin and Blood-Brain Barrier Disruption

BBB is mainly composed of capillary endothelial cells, pericytes, astrocytes, and vascular basilemma. Due to the tight connection between capillary endothelial cells, the cell gap is small. In the physiological state, most substances (such as plasma components, red blood cells, etc.) cannot pass the BBB except for a few lipid-soluble small molecules (Daneman and Barres, 2005; Zhao et al., 2015). In SAH, the expression of type IV collagen is significantly increased, which degrades the BBB basement membrane and is accompanied by the elevation of vascular endothelial growth factor, activation of endothelial cell apoptosis, resulting in the enhanced permeability of BBB (Yang and Rosenberg, 2011; Kanamaru and Suzuki, 2019; Li et al., 2020). Cerebral edema directly results from BBB dysfunction, which includes vasogenic and cytotoxic edema (Rosenberg, 1999; Sandoval and Witt, 2008). Vasogenic edema refers to blood flow from the vessels to brain tissue due to the apoptosis of endothelial cells and glial cells around blood vessels. The increase of intracranial pressure after SAH induces the decrease of CBF, which causes global cerebral ischemia, leading to the failure of the Na^+^ /K^+^ pump, and resulting in cytotoxic edema (Okada et al., 2020; Chen et al., 2021). Clinical studies have shown that about 8% of patients were found to have global cerebral edema after head CT examination upon admission, and another 12% of patients developed prominent cerebral edema within 6 days following SAH (Cahill et al., 2006). Severe cerebral edema often leads to increased intracranial pressure, acute cerebral ischemia, cerebral hernia, and death. Therefore, it is of great significance to protect the integrity of BBB and reduce the development of cerebral edema, aiming to improve the prognosis of patients (Michinaga and Koyama, 2015).

Pragmatic therapeutic strategies for brain edema such as acupuncture, osmotherapy, non-peptide vasopressin receptor antagonist, and calcium channel blockers are used in clinical practice (Rowland et al., 2019; Corry et al., 2020; Hinson et al., 2020; Guo et al., 2022). Although the above-mentioned treatment approaches have been well-studied and display partial protective effects in attenuating cerebral edema, a single therapy capable of inhibiting cerebral edema is yet to be found due to the complex mechanisms involved. Recently, experimental studies have shown that melatonin not only reduces cerebral edema but also protects the BBB by preventing the disruption of tight junction protein expression (ZO-1, occludin, and claudin-5), indicating that melatonin may be an effective alternative for alleviating brain edema after SAH (Ayer et al., 2008a,b; Chen et al., 2014a; Li et al., 2019; Liu et al., 2020). Additionally, melatonin can easily cross the BBB, while preserving BBB permeability and reducing brain edema (Ersahin et al., 2009).

### Melatonin and Oxidative Stress

Superoxide dismutase (SOD), glutathione peroxidase, catalase and other important antioxidant enzymes in brain tissue are down-regulated after SAH, leading to a decrease in antioxidant capacity. Meanwhile, vasospasm and cerebral edema caused by SAH lead to cerebral ischemia, resulting in the production of a large number of oxygen ions (O^2–^) and hydrogen peroxide (H_2_O_2_) (Yang et al., 2017; Shao et al., 2020). The high concentration of Fe^2+^ and Fe^3+^ produced by erythrocyte degradation can combine with H_2_O_2_ and O^2–^ by Fenton reaction to form hydroxyl radicals. Hydroxyl radicals are among the most toxic reactive oxygen species (ROS), which can directly damage neurovascular units and cause neurologic injury. In addition, free radicals-induced oxidative stress cause brain damage by promoting lipid peroxidation, protein degradation, and DNA destruction, resulting in neuronal apoptosis, endothelial cell damage, and BBB destruction. These changes result in severe brain injury and neurological deterioration after SAH (Lu et al., 2019; Wu et al., 2021). Therefore, the intervention of oxidative stress can inhibit the secondary cascade reaction of pathological changes and reduce subsequent brain damage (Zhang et al., 2014; Lin et al., 2021a).

Melatonin is a powerful antioxidant. Its antioxidant effects include direct scavenging of free radicals, stimulation of antioxidant enzyme activity and gene expression, stimulation of glutathione synthesis, reduction of electron leakage of mitochondrial electron transport chain, and reduction of cytokine production (Reiter et al., 2016; Galano and Reiter, 2018). Previous research has shown that lipopolysaccharides-induced hyperreactivity of vascular smooth muscle is mediated through enhanced release of ROS and prostanoids, and melatonin inhibits the vascular hyperreactivity *via* selective scavenging of ROS (Müller-Schweinitzer et al., 2004). Melatonin can cause a significant increase in brain glutathione (GSH) and superoxidase dismutase (SOD) content, as well as Na^+^-K^+^-ATPase activity and GSH/GSSG ratio, which is accompanied by significant decreases in ROS, malondialdehyde (MDA) levels, and myeloperoxidase (MPO) activity, thereby providing neuroprotection from EBI following SAH (Ersahin et al., 2009; Fang et al., 2009; Yang et al., 2018a). Besides, melatonin alleviates SAH-induced EBI by inhibiting the ROS-stimulated activation of the MST1 pathway (Shi et al., 2018), NLRP3 inflammasome (Cao et al., 2017), and SIRT3 pathway (Yang et al., 2018a). An *in vivo* and *in vitro* study demonstrated that H_2_O_2_ markedly upregulated the expression of H19, miR-675, and NGF, and downregulated let-7a and TP53 levels. These findings were reversed by melatonin treatment, revealing the potential antioxidant mechanisms of melatonin (Yang et al., 2018b). It is worth noting that Nrf2 is a global promoter of antioxidant response and has potential protective effects against post-SAH EBI. It has been shown that Nrf2-knockout animals have poorer outcomes in SAH. Melatonin can increase the effects of the antioxidant system by upregulating the expression of Nrf2 (Wang et al., 2012; Sun et al., 2018).

## Conclusion and Prospects

Melatonin is a neuroendocrine hormone that protects the central nervous system mainly through anti-vasospasm, anti-oxidative stress, anti-inflammatory response, anti-apoptosis and BBB protection. At present, the study of melatonin in SAH is mostly limited to animal and cell models, and lack of clinical evidence. So far, four clinical studies with small cohort of patients have explored the association between melatonin and SAH patients. Melatonin could decrease fatigue, but has no significant impact on depression and apathy post-stroke (Gilard et al., 2016). In addition, melatonin administration has no effect on delayed cerebral ischemia, but may reduce mortality of SAH (Lin et al., 2021b). Another prospective and observational study enrolls 169 aneurysmal SAH patients, to ascertain the relationship between endogenous melatonin level and neurological outcome post-SAH. The results indicate that higher level of serum melatonin is associated with poor outcome after SAH (Zhan et al., 2021). As many factors can affects the concentrations of serum melatonin such as the severity of brain injury, and rhythm of melatonin secretion, the accurate influence and changes of melatonin after SAH need to be studied. Neumaier et al. (2021) reports that there is a delayed upregulation of circulatory daytime melatonin levels after SAH, and higher concentration of melatonin is related with patients with anterior communicating artery aneurysms or poor clinical outcome, indicating the potential role of hypothalamic dysfunction. Further large-scale clinical trials are needed to verify its neuroprotective effect in SAH patients. Additionally, the effects of melatonin in patients with different degrees of injury and ages should be explored. Moreover, the secretion of melatonin in the body follows the circadian rhythm. The time of administration likely plays an essential role in achieving the optimal therapeutic effect, and needs further study. Furthermore, the drug dosage and administration interval of melatonin used in current studies vary greatly. The optimal dose and administration frequency should be determined to effectively improve the therapeutic effect. Finally, melatonin regulates biological rhythms, and a large proportion of SAH patients have sleep disorders. Determining the therapeutic strategy of melatonin for this population is worth exploring.

## Author Contributions

CX and ZH conceived the perspective of the work and searched the literature and drafted the manuscript. JL critically revised the article. All authors contributed to and approved to the final manuscript.

## Conflict of Interest

The authors declare that the research was conducted in the absence of any commercial or financial relationships that could be construed as a potential conflict of interest.

## Publisher’s Note

All claims expressed in this article are solely those of the authors and do not necessarily represent those of their affiliated organizations, or those of the publisher, the editors and the reviewers. Any product that may be evaluated in this article, or claim that may be made by its manufacturer, is not guaranteed or endorsed by the publisher.
